# Supplementary material for: Patient-reported outcomes and neurotoxicity markers in patients treated with bispecific LV20.19 CAR T cell therapy
Source: Commun Med (Lond). 2022 May 12;2:49. doi: 10.1038/s43856-022-00116-5 (PMC9098435; doi:10.1038/s43856-022-00116-5)
Supplement: Supplementary file 3 — Supplemental Materials [file 43856_2022_116_MOESM3_ESM.pdf]

Supplemental Table 1. Clinical details for participants who developed neurotoxicity

NTX= neurotoxicity; toci = tocilizumab; dex= dexamethasone; IT = intrathecal;

MTX=methotrexate; ARA-C = cytarabine, MCL=mantle cell lymphoma; CSF=cerebrospinal fluid

Participant No.	Max NTX Grade	NTX Onset Day	Treatment	Duration of NTX	Presentation of Neurotoxicity
**08**	1	9	Toci x1 dose<br>Dex taper	2 days	Eye pressure, headache, confusion, floaters, hallucinations, word finding difficulties
**17**	3	6	Toci x 1 dose<br>Dex taper<br>Pulse Solumedrol IT MTX, ARA-C, hydrocortisone	24 days	Peripheral neuropathy, tremors (R foot drop), somnolence, delirium, dysphasia
**15**	3	0	Siltuximab<br>Dex taper	1st NTX (gr3): 2 days<br><br>2nd NTX (gr2): 13 days, diagnosed with MCL involvement of CSF	Disorientation, somnolence, confusion
**20**	1	6	Toci x 1 dose<br>Dex taper	2 days	Confusion, headache, mild slowness in speech
**26**	4	1	Toci x 1 dose<br>Dex taper<br>Pulse Solumedrol x 3 doses<br>IT hydrocortisone	8 days	Confusion/tremors, seizure, lethargy, delirium, somnolence, intubated for airway protection
