# Supplementary material for: Patient-reported outcomes and neurotoxicity markers in patients treated with bispecific LV20.19 CAR T cell therapy
Source: Commun Med (Lond). 2022 May 12;2:49. doi: 10.1038/s43856-022-00116-5 (PMC9098435; doi:10.1038/s43856-022-00116-5)
Supplement: Supplementary file 5 — Reporting Summary [file 43856_2022_116_MOESM5_ESM.pdf]

nature research

Corresponding author(s): Jennifer M. Knight
Last updated by author(s): Sep 28, 2021

# Reporting Summary

Nature Research wishes to improve the reproducibility of the work that we publish. This form provides structure for consistency and transparency in reporting. For further information on Nature Research policies, see our Editorial Policies and the Editorial Policy Checklist.

## Statistics

For all statistical analyses, confirm that the following items are present in the figure legend, table legend, main text, or Methods section.

| n/a | Confirmed | |
|---|---|---|
| ☐ | ☒ | The exact sample size ($n$) for each experimental group/condition, given as a discrete number and unit of measurement |
| ☒ | ☐ | A statement on whether measurements were taken from distinct samples or whether the same sample was measured repeatedly |
| ☐ | ☒ | The statistical test(s) used AND whether they are one- or two-sided<br>*Only common tests should be described solely by name; describe more complex techniques in the Methods section.* |
| ☐ | ☒ | A description of all covariates tested |
| ☐ | ☒ | A description of any assumptions or corrections, such as tests of normality and adjustment for multiple comparisons |
| ☐ | ☒ | A full description of the statistical parameters including central tendency (e.g. means) or other basic estimates (e.g. regression coefficient) AND variation (e.g. standard deviation) or associated estimates of uncertainty (e.g. confidence intervals) |
| ☐ | ☒ | For null hypothesis testing, the test statistic (e.g. $F$, $t$, $r$) with confidence intervals, effect sizes, degrees of freedom and $P$ value noted<br>*Give P values as exact values whenever suitable.* |
| ☒ | ☐ | For Bayesian analysis, information on the choice of priors and Markov chain Monte Carlo settings |
| ☒ | ☐ | For hierarchical and complex designs, identification of the appropriate level for tests and full reporting of outcomes |
| ☒ | ☐ | Estimates of effect sizes (e.g. Cohen's $d$, Pearson's $r$), indicating how they were calculated |

*Our web collection on statistics for biologists contains articles on many of the points above.*

## Software and code

Policy information about availability of computer code

| | |
|---|---|
| Data collection | Data was collected utilizing OnCore Enterprise Database and Microsoft Excel |
| Data analysis | Analyses were performed using SAS 9.4 (SAS Institute, Cary, NC), Stata 13.1 (Stata Corp, College Station, TX), and GraphPad Prism v8 software. P values <0.05 were considered as significant. For flow cytometry analyses, BD FACSDiva v8.01 and FlowJo v8.7 were used. |

For manuscripts utilizing custom algorithms or software that are central to the research but not yet described in published literature, software must be made available to editors and reviewers. We strongly encourage code deposition in a community repository (e.g. GitHub). See the Nature Research guidelines for submitting code & software for further information.

## Data

Policy information about availability of data

All manuscripts must include a data availability statement. This statement should provide the following information, where applicable:

- Accession codes, unique identifiers, or web links for publicly available datasets
- A list of figures that have associated raw data
- A description of any restrictions on data availability

The LV20.19 sequence is available in Shah et al. (2020). All requests for raw and analyzed data and materials are will be promptly reviewed by the Medical College of Wisconsin and Lentigen Technology to verify whether the request is subject to any intellectual property, confidentiality obligations. Patient-related data not included in the paper were generated as part of clinical trials and may be subject to patient confidentiality. Any data and materials that can be shared will be released via a material transfer agreement. All other data that support the findings of this study will be provided by the corresponding author upon reasonable request when possible.



April 2020

# Field-specific reporting

Please select the one below that is the best fit for your research. If you are not sure, read the appropriate sections before making your selection.

☒ Life sciences ☐ Behavioural & social sciences ☐ Ecological, evolutionary & environmental sciences

For a reference copy of the document with all sections, see nature.com/documents/nr-reporting-summary-flat.pdf

# Life sciences study design

All studies must disclose on these points even when the disclosure is negative.

**Sample size**

15

**Data exclusions**

4 patients of the original study cohort did not achieve the target LV20.19 CAR T-cell dose and were excluded from the primary analysis. These patients were replaced as predefined in the clinical protocol for not achieving target dose.
6 patients did not have Quality of Life patient reported outcomes documented and were excluded from the analysis.
1 patient did not survive until day 28 assessment, so their original data was excluded.

**Replication**

As patients were treated with a study intervention, we cannot replicate the cell infusion in individual patients. The datasets were analyzed and findings of the statistical analysis can be replicated.

**Randomization**

As a Phase 1, safety study, randomization is not appropriate. Patients were enrolled consecutively at the cohort with the highest available dose.

**Blinding**

No blinding was used in this open-label study

# Reporting for specific materials, systems and methods

We require information from authors about some types of materials, experimental systems and methods used in many studies. Here, indicate whether each material, system or method listed is relevant to your study. If you are not sure if a list item applies to your research, read the appropriate section before selecting a response.

## Materials & experimental systems

| n/a | Involved in the study | |
|---|---|---|
| ☒ | ☐ | Antibodies |
| ☒ | ☐ | Eukaryotic cell lines |
| ☒ | ☐ | Palaeontology and archaeology |
| ☒ | ☐ | Animals and other organisms |
| ☐ | ☒ | Human research participants |
| ☐ | ☒ | Clinical data |
| ☐ | ☒ | Dual use research of concern |

## Methods

| n/a | Involved in the study | |
|---|---|---|
| ☒ | ☐ | ChIP-seq |
| ☐ | ☒ | Flow cytometry |
| ☒ | ☐ | MRI-based neuroimaging |

## Human research participants

Policy information about studies involving human research participants

**Population characteristics**

Adult patients 18 years of age or older with relapsed refractory B-cell non-Hodgkin lymphoma or chronic lymphocytic leukemia were included in this clinical trial. The median age of patients was 61 years (range 38-72 years) and most patients were male (14/15).

**Recruitment**

Patients were recruited from the Froedtert Hospital and Medical College of Wisconsin Grace Clinic which include specialists in hematological malignancies. As a tertiary referral center, patients who are identified by local referring doctors as potential candidates for advanced therapies are sent which may lead to a selection bias of healthier and younger patients. Similarly there may be a self-selection bias that occurs from patients who self refer themselves to this institution. Socioeconomic status and educational status may also impact the profile of patients seen at an academic center.

**Ethics oversight**

This study was approved by the Medical College of Wisconsin and Froedtert Hospital Institutional Review Board and FDA under IND 17518. All patients signed informed consent prior to any study related procedures. A copy of the informed consent document was provided to each patient.

Note that full information on the approval of the study protocol must also be provided in the manuscript.

# Clinical data

Policy information about clinical studies

All manuscripts should comply with the ICMJE guidelines for publication of clinical research and a completed CONSORT checklist must be included with all submissions.

| | |
|---|---|
| Clinical trial registration | Clinicatrials.gov=NCT03019055 |
| Study protocol | Study protocol will be provided as part of this submission and reported apart of Shah et al. 2020 |
| Data collection | Patients were recruited following IRB approval in August 2017 until last patient accrual in July 2019. Data was collected from patients during their outpatient visits, inpatient hospitalization for CAR T-cell treatment, and at designated follow-up time points. Data was collected by the Medical College of Wisconsin Clinical Trials Office research coordinators and retained internally utilizing Oncore database. |
| Outcomes | This is a Phase 1 safety and feasibility study. The primary endpoint was identification of a safe cell dose of LV20.19 CAR T-cells. Safety was assessed by the presence of dose limiting toxicities (DLT) during the first 28-day after LV20.19 CAR T-cell infusion. A DLT<33% was required to deem a dose-level safe and for escalation and the DLT definition is available in the clinical protocol. Secondary endpoints included response rates, duration of response, progression free survival, overall survival, and Quality of Life measurements such as depression, fatigue, and anxiety levels. A second primary endpoint was to determine the feasibility of CliniMACS Prodigy device for point-of-care LV20.19 CAR T-cell production and feasibility was set at a >75% successful LV20.19 CAR T-cell production rate. |

# Dual use research of concern

Policy information about dual use research of concern

## Hazards

Could the accidental, deliberate or reckless misuse of agents or technologies generated in the work, or the application of information presented in the manuscript, pose a threat to:

| No | Yes | |
|---|---|---|
| ☒ | ☐ | Public health |
| ☒ | ☐ | National security |
| ☒ | ☐ | Crops and/or livestock |
| ☒ | ☐ | Ecosystems |
| ☒ | ☐ | Any other significant area |

## Experiments of concern

Does the work involve any of these experiments of concern:

| No | Yes | |
|---|---|---|
| ☒ | ☐ | Demonstrate how to render a vaccine ineffective |
| ☒ | ☐ | Confer resistance to therapeutically useful antibiotics or antiviral agents |
| ☒ | ☐ | Enhance the virulence of a pathogen or render a nonpathogen virulent |
| ☒ | ☐ | Increase transmissibility of a pathogen |
| ☒ | ☐ | Alter the host range of a pathogen |
| ☒ | ☐ | Enable evasion of diagnostic/detection modalities |
| ☒ | ☐ | Enable the weaponization of a biological agent or toxin |
| ☒ | ☐ | Any other potentially harmful combination of experiments and agents |

# Flow Cytometry

## Plots

Confirm that:

☒ The axis labels state the marker and fluorochrome used (e.g. CD4-FITC).

☒ The axis scales are clearly visible. Include numbers along axes only for bottom left plot of group (a 'group' is an analysis of identical markers).

☒ All plots are contour plots with outliers or pseudocolor plots.

☒ A numerical value for number of cells or percentage (with statistics) is provided.

## Methodology

| | |
|---|---|
| Sample preparation | All cells analyzed by flow cytometry were from human subjects enrolled on the study. Cell samples analyzed included the following: apheresis collections, CD4- and CD8-enriched T cells, in-process samples from CAR-T manufacturing runs, final CAR-T cell products, biopsy samples, and peripheral blood mononuclear cells. Peripheral blood mononuclear cells were isolated using Biocoll (similar to Ficoll) cell separation solution (Millipore Sigma). Biopsy materials were processed into single cell suspensions by mechanical disruption. All cells were analyzed the same day they were stained. The only samples that were fixed were those stained for intracellular IFN-gamma expression. |
| Instrument | Becton Dickinson (BD) FACSCanto II flow cytometer. |
| Software | BD FACSDiva software or FlowJo software was used for analysis of flow cytometry data. |
| Cell population abundance | We did not do any flow cytometric sorting. However, as part of the CAR-T cell manufacturing process, T cells were enriched within the CliniMACS Prodigy device by immunomagnetic sorting using a combination of anti-CD4- and anti-CD8-conjugated MACS beads (Miltenyi Biotec). A sample of the the T cell-enriched cells were analyzed for purity by flow cytometry using a combination of markers including CD3, CD4 and CD8. |
| Gating strategy | The typical gating strategy for each flow cytometry analysis was the following: (a) set a debris-free gate based on a FSC-A by SSC- A plot; (b) on the debris-free gated cells, the viable cells were gated (7-AAD-negative); (c) examine expression of specific markers based on the gated viable cells. For apheresis products and peripheral blood mononuclear cells, when T or B cells were analyzed, a viable lymphocyte gate was set. Boundaries between positive and negative staining cell populations were based on negative and positive controls. These controls consisted of unstained controls and non-transduced cell controls. |

☐ Tick this box to confirm that a figure exemplifying the gating strategy is provided in the Supplementary Information.
